# Comparison of Causal and Non-causal Strategies for the Assessment of Baroreflex Sensitivity in Predicting Acute Kidney Dysfunction After Coronary Artery Bypass Grafting

**DOI:** 10.3389/fphys.2019.01319

**Published:** 2019-10-18

**Authors:** Vlasta Bari, Emanuele Vaini, Valeria Pistuddi, Angela Fantinato, Beatrice Cairo, Beatrice De Maria, Laura Adelaide Dalla Vecchia, Marco Ranucci, Alberto Porta

**Affiliations:** ^1^Department of Cardiothoracic, Vascular Anesthesia and Intensive Care, IRCCS Policlinico San Donato, Milan, Italy; ^2^Department of Biomedical Sciences for Health, University of Milan, Milan, Italy; ^3^IRCCS Istituti Clinici Scientifici Maugeri, Milan, Italy

**Keywords:** heart rate variability, arterial pressure, autonomic nervous system, cardiovascular control, adverse outcome, intensive care unit, cardiac surgery, propofol anesthesia

## Abstract

Coronary artery bypass graft (CABG) surgery may lead to postoperative complications such as the acute kidney dysfunction (AKD), identified as any post-intervention increase of serum creatinine level. Cardiovascular control reflexes like the baroreflex can play a role in the AKD development. The aim of this study is to test whether baroreflex sensitivity (BRS) estimates derived from non-causal and causal approaches applied to spontaneous systolic arterial pressure (SAP) and heart period (HP) fluctuations can help in identifying subjects at risk of developing AKD after CABG and which BRS estimates provide the best performance. Electrocardiogram and invasive arterial pressure were acquired from 129 subjects (67 ± 10 years, 112 males) before (PRE) and after (POST) general anesthesia induction with propofol and remifentanil. Subjects were divided into AKDs (*n* = 29) or no AKDs (noAKDs, *n* = 100) according to the AKD development after CABG. The non-causal approach assesses the transfer function from the HP-SAP cross-spectrum in the low frequency (LF, 0.04–0.15 Hz) band. BRS was estimated according to three strategies: (i) sampling of the transfer function gain at the maximum of the HP-SAP squared coherence in the LF band; (ii) averaging of the transfer function gain in the LF band; (iii) sampling of the transfer function gain at the weighted central frequency of the spectral components of the SAP series dropping in the LF band. The causal approach separated the two arms of cardiovascular control (i.e., from SAP to HP and *vice versa*) and accounted for the confounding influences of respiration via system identification and modeling techniques. The causal approach provided a direct estimate of the gain from SAP to HP by observing the HP response to a simulated SAP rise from the identified model structure. Results show that BRS was significantly lower in AKDs than noAKDs during POST regardless of the strategy adopted for its computation. Moreover, all the BRS estimates during POST remained associated with AKD even after correction for demographic and clinical factors. Non-causal and causal BRS estimates exhibited similar performances. Baroreflex impairment is associated with post-CABG AKD and both non-causal and causal methods can be exploited to improve risk stratification of AKD after CABG.

## Introduction

Cardiac baroreflex (BR) is a regulatory mechanism aiming at maintaining the physiological homeostasis by adjusting heart period (HP) in response to arterial pressure (AP) variations (Smyth et al., 1969; Laude et al., 2004). The efficiency of this reflex is generally assessed by computing the baroreflex sensitivity (BRS), quantifying the magnitude of HP changes following a unit variation of AP (Vanoli and Adamson, 1994; Pinna et al., 2015).

The gold standard strategy to characterize BR is the invasive pharmacological method (Smyth et al., 1969) that evaluates the slope of the response of HP to a pharmacologically induced increase or decrease in systolic AP (SAP), referred to as BR sensitivity (BRS). Non-invasive strategies to assess BRS exist and are based on the analysis of the spontaneous variability of HP and SAP in time domain (Bertinieri et al., 1985; Westerhof et al., 2004; Bauer et al., 2010; Muller et al., 2012; De Maria et al., 2018), or frequency domain (Robbe et al., 1987; Pagani et al., 1988; Saul et al., 1991; Porta et al., 2000, 2013; Faes et al., 2004; Pinna et al., 2017) or using identification procedures estimating parameters of mathematical models (Baselli et al., 1994; Patton et al., 1995; Porta et al., 2000; Nollo et al., 2001; Xiao et al., 2005; Porta et al., 2013).

Among the different methodologies to assess BRS, the non-causal method in the frequency domain has been the most frequently exploited one (Robbe et al., 1987; Pagani et al., 1988; Saul et al., 1991; Porta et al., 2000, 2013; Faes et al., 2004; Pinna et al., 2017). The non-causal approach in the frequency domain is grounded on the computation of the cross-spectrum between the HP and SAP variability series and on the estimation of the transfer function directly from it. Then, once obtained the HP-SAP transfer function, a strategy is needed to convert the gain function into a single number representing the BRS. Non-causal frequency domain BRS markers are mostly assessed in the low frequency (LF, from 0.04 to 0.15 Hz) because in this band the prerequisites for their safe computation, namely the high HP-SAP association and HP variations lagging behind SAP ones are more likely to be fulfilled (de Boer et al., 1985). Moreover, the baroreflex origin of the LF oscillations detected in HP series has been repeatedly suggested as a consequence of resonance properties of baroreflex control loop and/or the latency of the baroreflex circuit (De Boer et al., 1987; Baselli et al., 1994; Goldstein et al., 2011).

Baroreflex sensitivity estimates derived from the causal closed loop approach (Baselli et al., 1994; Xiao et al., 2005; Porta et al., 2013) have recently gained attention. The main features of this class of BRS markers is that directionality of the HP-SAP dynamical interactions and their closed loop nature are explicitly accounted by the model structure underlying their computation. Since the two characteristics are neglected by more traditional non-causal frequency domain approaches, causal closed loop BRS markers might have additional advantage in clinical applications. Moreover, the additional advantage of the causal closed loop BRS estimates is the possibility of accounting for the presence of confounding factors, such as respiration (RESP) (Porta et al., 2012), contaminating both HP and SAP variability.

The impairment of BR has a clinical value given that low BRS values are associated to adverse outcome in several pathological conditions. As a matter of fact, low BRS has a remarkable predictive power of adverse events in chronic heart failure (Gouveia et al., 2015; Pinna et al., 2017), myocardial infarction (Landolina et al., 1997; La Rovere et al., 1998; De Ferrari et al., 2007), diabetes (Gerritsen et al., 2001) and hypertension (Collier et al., 2001; Gerritsen et al., 2001). More recently, BRS estimates have been found to be useful in stratifying the risk of adverse events and morbidity after major surgery (Toner et al., 2016; Ranucci et al., 2017) and the inability to cope with increased SAP variability has been correlated with a greater risk in critical care unit (ICU) (Porta et al., 2018). Acute kidney dysfunction (AKD) is one of the major complications after coronary artery bypass graft (CABG) surgery (Ranucci et al., 2017). Since AKD increases early and long-term mortality (Liotta et al., 2014) and it is associated to cardiovascular complications such as heart failure (Holzmann et al., 2013), the prevention of AKD after CABG would improve the patient’s prognosis. Moreover, it would reduce patient’s ICU stay and, consequently, hospitalization costs.

The use of BRS markers in predicting AKD after CABG requires the optimization of the technique for the computation of BRS. Indeed, the perioperative evaluation of BRS in patients undergoing CABG is a challenging issue because they usually feature an impaired BR regulation and the concomitant presence of pharmacological therapy (Porta et al., 2013; Bari et al., 2018). Therefore, the aim of this work is to check the performance of non-causal and causal strategies for the BRS quantification in differentiating patients who developed AKD after CABG from the ones who did not (noAKD). We consider three non-causal strategies to compute BRS based on cross-spectrum estimation from spontaneous HP and SAP variability: (i) sampling the transfer function gain at the maximum of the squared coherence function (*K*^2^) in the LF band (Bari et al., 2018); (ii) averaging the transfer function gain in the LF band (Pinna et al., 2017); (iii) sampling the transfer function gain at the weighted central frequency of the SAP spectral components dropping in the LF band (Porta et al., 2000, 2013). These three non-causal markers were compared to a causal BRS estimate accounting for the closed loop HP-SAP dynamical interactions and RESP influences (Baselli et al., 1994; Porta et al., 2013). Each technique was tested in patients scheduled for CABG before and after the induction of propofol general anesthesia.

## Materials and Methods

### Non-causal Open Loop Assessment of BRS in the Frequency Domain

The non-causal approach is based on the estimation of the traditional input-output relation via the cross-spectrum between two series (Saul et al., 1991; Pinna et al., 2002). Being based on the cross-spectrum, this is an open loop approach that hides the closed loop structure of the interactions (Porta et al., 2002). Cross-spectrum was estimated through a parametric approach based on the computation of the coefficients of the bivariate autoregressive model assessed over HP and SAP (Porta et al., 2000). The model order was fixed to 10. The coefficients of the model were identified through least squares approach (Baselli et al., 1997). The transfer function was estimated as the ratio of the cross-spectrum computed from SAP to HP to the power spectrum of SAP. The BRS function was computed in the frequency domain as the modulus of the transfer function in the LF band, namely from 0.04 to 0.15 Hz (Task Force of the European Society of Cardiology, and the North American Society of Pacing and Electrophysiology, 1996). The squared HP-SAP coherence *K*^2^ was computed as the ratio between the squared modulus of the HP-SAP cross-spectrum divided by the product of the two spectra of HP and SAP. This function was labeled as *K*^2^ and ranged between 0 and 1, with 0 indicates null correlation and 1 maximum correlation.

### Strategies to Derive the BRS Marker From the BRS Function

**FIGURE 1:**
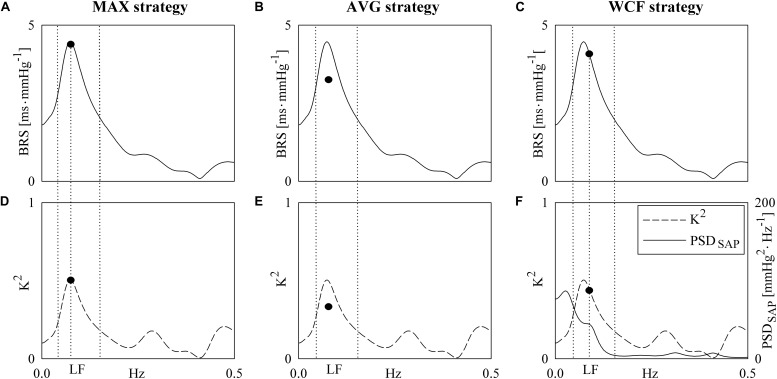
The line graphs show examples of computation of the BRS markers. They are derived from HP and SAP series recorded from the same subject during PRE. The first strategy samples the BRS function **(A)** in the LF band at the maximum of *K*^2^ **(D)**. The sampling of the BRS function and *K*^2^ is marked with a solid circle in both **(A)** and **(D)**. The second strategy averages the BRS function in the LF band. The average of the BRS and the average of *K*^2^ is indicated with a solid circle in **(B)** and **(E)**. The third strategy samples the BRS function **(C)** at the WCF of the spectral components of SAP power spectral density PSD_SAP_ (**F**, solid line) detected in LF band. The sampling of the BRS function **(C)** and *K*^2^ (**F**, dashed line) is marked with a solid circle. The frequency in correspondence of the sampling was indicated as vertical dotted lines as well as the inferior and superior limits of the LF band.

A single BRS value was derived from the BRS function according to three different strategies. The first strategy (Figures 1A,D), denoted as the MAX strategy, computed the BRS marker as the sampling of the BRS function at the maximum of the *K*^2^ in LF band (Bari et al., 2018). The index was indicated as BRS_MAX_. The correspondent peak of the *K*^2^ was also stored and labeled as *K*^2^_MAX_. The second approach took the average of the BRS function in the LF band (Figures 1B,E) (Pinna et al., 2017). This approach will be referred to as the AVG strategy. The index was indicated as BRS_AVG_. Similarly, *K*^2^ was averaged in the LF band and this average was indicated as *K*^2^_AVG_. The third strategy assessed BRS (Figures 1C,F) as the sampling of the BRS function at the weighted central frequency (WCF) of SAP, namely at the average central frequency of spectral components of SAP series dropping in the LF band computed using their power as weights (Porta et al., 2000, 2013). The spectral decomposition technique was applied to obtain SAP spectral components, their central frequency and power (Baselli et al., 1997). This approach will be referred to as the WCF strategy. The BRS marker was indicated as BRS_WCF_. *K*^2^ was sampled at WCF as well and the value was indicated as *K*^2^_WCF_. *K*^2^ markers were dimensionless, while BRS indexes were expressed in ms⋅mmHg^–1^. It is worth noting that, while MAX and AVG approaches can be applied in the 100% of the subjects in any experimental condition, the WCF one can be performed only whether at least one spectral component with central frequency dropping in the LF band was detected in the power spectrum of SAP variability series. By definition, BRS_MAX_, BRS_AVG_ and BRS_WCF_ were larger than 0.

### Causal Closed Loop Assessment of BRS

The gains along the two arms of the HP-SAP closed loop control (i.e., the baroreflex feedback pathway from SAP to HP and the mechanical feedforward arm from HP to SAP) were estimated according to a causal linear trivariate model describing the HP-SAP dynamical interactions and considering RESP as an exogenous input acting on both series (Baselli et al., 1994; Porta et al., 2000). In particular, the current HP and SAP were described according to an autoregressive model with exogenous input that combines previous samples of the same series with previous, and eventually present, values of the other series present in the set formed by HP, SAP, RESP with a sample of a white random noise. Being the model structure fully exploited to disentangle the baroreflex feedback pathway from the mechanical feedforward one and considering the directional structure of the model blocks (i.e., the output depends on past, and eventually present, values of the input), the approach is in closed loop and causal. Since RESP was considered exogenous to both HP and SAP time series, RESP dynamic was modeled via the linear combination of its past values plus a sample of a random white noise. All regressions had the same order optimized in the range from 4 to 14 via the Akaike information criterion for multivariate processes. Further details on the procedures followed to estimate the coefficients of the linear regressions and optimization of the model order can be found in Baselli et al. (1997), Porta et al. (2013). The BR gain was computed by feeding the block representing the dynamical relation from SAP to HP with an artificial SAP ramp of unit slope simulating a SAP rise. The slope of the corresponding HP response computed over the first 15 samples was then taken as an estimate of BRS, labeled as BRS_SAP__→__HP_, and expressed in ms⋅mmHg^–1^. The gain of the mechanical feedforward pathway was estimated as the first coefficient of the dynamical relation from HP to SAP. It was labeled as a_HP__→__SAP_(1) and expressed in mmHg⋅s^–1^. Both BRS_SAP__→__HP_ and a_HP__→__SAP_(1) can be smaller than 0.

### Experimental Protocol

One-hundred twenty-nine patients (67 ± 10 years, age from 43 to 86 years, 112 males) scheduled for elective, or urgent, CABG surgery at the Department of Cardiothoracic and Vascular Anesthesia and Intensive Care of the IRCCS Policlinico San Donato, San Donato Milanese, Milan, Italy, were enrolled for this study. The study was performed in keeping with the Declaration of Helsinki for research studies involving humans and, before participating, subjects signed a written informed consent. The study was approved by the ethical committee in charge at the IRCCS Policlinico San Donato.

Inclusion criteria were sinus rhythm, age over 18 years, absence of previous kidney dysfunction and of autonomic nervous system pathology. Electrocardiogram (ECG) and invasive AP, measured at the radial artery, were acquired directly from patient’s monitor with an analog-to-digital board (National Instruments, Austin, TX, United States) connected to a laptop for 10 min before (PRE) and after (POST) the induction of general anesthesia performed with propofol and remifentanil. About 1 h before the first acquisition, patients were treated with an intramuscular injection of 0.5 mg of atropine and 100 μg of fentanyl. Anesthesia was induced with an intravenous bolus of 1.5 mg⋅kg^–1^ of propofol and 0.2 μg⋅kg^–1^⋅min^–1^ of remifentanil according to the standard practice of our institute and was then maintained by infusion at a rate of 3 mg⋅kg^–1^⋅h^–1^ and from 0.05 to 0.5 μg⋅kg^–1^⋅min^–1^ respectively. Subjects breathed spontaneously during PRE and were mechanically ventilated at a rate from 12 to 16 breaths per minute during POST, inhaling a mixture of 1:1 of oxygen and air. Patients were followed during their stay in ICU after CABG surgery and their serum creatinine level was monitored. AKD was defined as any postoperative increase of serum creatinine level from preoperative values in the first 48 h after surgery (Ranucci et al., 2017). Patients were then divided in two groups, defined as AKD (*n* = 29, age 68.7 ± 10.6, 24 males) and noAKD (*n* = 100, age 66.0 ± 9.4, 88 males), according to whether the occurrence of AKD after CABG surgery was observed or not.

### Beat-to-Beat Series Extraction and Time Domain Indexes

From ECG and AP signals, beat-to-beat variability series were extracted. HP was measured as the temporal distance between two R-wave peaks on the ECG. SAP was taken as the maximum of AP inside the HP and diastolic AP (DAP) as the minimum of AP following SAP. The amplitude of the first QRS complex (from baseline to apex) was utilized as an ECG-derived RESP series (Porta et al., 1998). Series lasting 250 beats were extracted during PRE and POST and they were manually inspected and corrected in case of missing beats or misdetections. The effect of ectopic beats was limited via linear interpolation using the most adjacent values of HP, SAP and DAP unaffected by ectopies. Corrections never exceeded 5% of total beats utilized for the analysis. Time domain indexes as mean and variance of HP, SAP and DAP were calculated. They were labeled as μ_HP_, σ^2^_*HP*,_ μ_SAP_, σ^2^_SAP_, μ_DAP_ and σ^2^_DAP_, and expressed respectively in ms, ms^2^, mmHg, mmHg^2^, mmHg, mmHg^2^.

### Statistical Analysis

Unpaired *t*-test, or Mann–Whitney rank sum test when appropriate, was applied over demographic and clinical variables to test their difference between noAKD and AKD groups. χ^2^ test was used in case of dichotomous variables. Two-way repeated measures analysis of variance (one factor repetition, Holm–Sidak test for multiple comparisons) was performed over cardiovascular control parameters to assess differences between groups (i.e., noAKD and AKD) assigned the experimental condition (i.e., PRE or POST) and between conditions assigned the group of individuals.

In the multivariate logistic regression model built over demographic and clinical factors capable of discriminating the two groups with *p* < 0.1 the cardiovascular variability markers were introduced one by one. Regression coefficient, odds ratio, 95% confidence interval and type I error probability *p* of the multivariate logistic regression model were evaluated to assess the degree of association of cardiovascular variability markers with the outcome accounting for the demographic and clinical factors. For the variability markers that remained associated with the outcome with *p* < 0.05, a receiver operating characteristic (ROC) curve was calculated at the univariate level and the area under the ROC curve (AUC) was assessed. The best combination of sensitivity and specificity was found according to the Youden index for each variability parameter remaining significantly associated with the outcome and the negative predictive value (NPV) and positive predictive value (PPV) were consequently assessed. Then, a ROC curve was built using all the clinical parameters that remained associated with the outcome with *p* < 0.05 alone or in combination with every single cardiovascular variability marker that remained associated with the outcome. The performance of the discrimination between AKD and noAKD group of the multivariate logistic regression models was evaluated via the AUC. Statistical analyses were carried out using commercial statistical software (Sigmaplot version 14.0, Systat, Inc., Chicago, IL, United States and IBM SPSS Statistics version 22.0, IBM, Armonk, NY, United States). A *p* < 0.05 was deemed as significant for all the analyses.

## Results

Table 1 summarizes clinical and demographic parameters of noAKD and AKD subjects. Only hematocrit (HTC) was lower in patients developing AKD post-surgery. The preoperative serum creatinine level was not different between groups but the type I error probability *p* was below the value set to include this parameter in the multivariate logistic regression model (i.e., 0.1). As a consequence of the outcome, patients developing AKD had a longer mechanical ventilation time and stay in intensive care unit.

**TABLE 1:** Clinical and demographic markers in noAKD and AKD subjects.

**Marker**	**noAKD (*n* = 100)**	**AKD (*n* = 29)**	***p***
Age [years]	66.0 ± 9.4	68.7 ± 10.6	0.21
Gender [male]	88 (88)	24 (83)	0.32
Weight [kg]	78.6 ± 14.9	78.1 ± 17.4	0.88
BMI [kg⋅m^–2^]	28.2 ± 14.7	27.2 ± 4.94	0.70
Congestive heart failure	3 (3)	2 (7)	0.31
Recent myocardial infarction	14 (14)	4 (14)	0.55
LVEF [%]	52.8 ± 11.9	52 ± 10.3	0.75
Diabetes	29 (29)	11 (38)	0.24
COPD	7 (7)	3 (10)	0.40
Serum creatinine [mg⋅dl^–1^]	1.0 ± 0.3	1.2 ± 0.87	0.08
Hypertension	62 (62)	22 (76)	0.12
Previous cerebrovascular accident	7 (7)	2 (7)	0.67
HCT [%]	39.6 ± 3.8	36.4 ± 4.6	<0.001
Catecholamine administration	14 (14)	3 (33)	0.15
ACE inhibitors	29 (29)	11 (38)	0.24
Beta-blockers	57 (57)	17 (59)	0.53
Calcium antagonists	7 (7)	0 (0)	0.16
Amiodarone	8 (8)	4 (14)	0.27
Combined intervention	6 (6)	2 (7)	0.57
Logistic EuroSCORE	1.8 ± 1.8	2.3 ± 1.2	0.12
CPB time [minutes]	63.3 ± 20.7	66.8 ± 26.8	0.45
Nadir temperature on CPB [°C]	32.9 ± 0.8	32.9 ± 0.9	0.98
Mechanical ventilation time [hours]	12.1 ± 1.6	17.8 ± 11.5	<0.001
ICU stay [days]	2.0 ± 1.6	3.0 ± 2.2	0.001
Hospital stay [days]	7.7 ± 2.5	8.0 ± 3.2	0.61

*BMI, body mass index; LVEF, left ventricular ejection fraction; COPD, chronic obstructive pulmonary disease; HCT, hematocrit; ACE, angiotensin converting enzyme; EuroSCORE, European System for Cardiac Operative Risk Evaluation; CPB, cardiopulmonary bypass; ICU, intensive care unit; *p*, type I error probability. Continuous data are presented as mean ± standard deviation and categorical data as number (percentage).*

Table 2 shows results of time domain parameters assessed in noAKD and AKD subjects during PRE and POST. The cardiovascular control depression induced by general anesthesia was evident. In fact, an increase of μ_HP_ during POST could be observed in both noAKD and AKD groups as well as a concomitant reduction of μ_SAP_, μ_DAP_ and σ^2^_HP_. σ^2^_SAP_ was significantly reduced in POST only in noAKDs. Only σ^2^_HP_ and σ^2^_SAP_ were able to separate AKD from noAKD group: indeed, during PRE σ^2^_HP_ and σ^2^_SAP_ were lower in AKDs than noAKDs. σ^2^_DAP_ was similar in both groups irrespective of the experimental condition.

**TABLE 2:** Time domain parameters in noAKD and AKD patients during PRE and POST.

**Marker**	**PRE**		**POST**	
	**noAKD**	**AKD**	**noAKD**	**AKD**
μ_HP_ [ms]	936.7 ± 147.0	922 ± 124.0	1112.2 ± 152.7^∗^	1091.0 ± 216.9^∗^
σ^2^_HP_ [ms^2^]	1709.9 ± 1473.4	1016.8 ± 1110.7^§^	714.9 ± 996.4^∗^	333.5 ± 289.8^∗^
μ_SAP_ [mmHg]	160.2 ± 27.7	170.4 ± 30.5	108.1 ± 18.6^∗^	109.0 ± 25.7^∗^
σ^2^_SAP_ [mmHg^2^]	34.5 ± 49.3	20.5 ± 12.4^§^	17.3 ± 19.3^∗^	12.1 ± 7.4
μ_DAP_ [mmHg]	78.0 ± 17.6	72.6 ± 10.4	61.5 ± 9.9^∗^	59.7 ± 10.5^∗^
σ^2^_DAP_ [mmHg^2^]	64.7 ± 181.9	25.6 ± 80.7	67.8 ± 251.7	8.5 ± 9.4

*HP, heart period; AP, arterial pressure; SAP, systolic AP; DAP, diastolic AP; μ_*HP*_, HP mean; σ^2^_*HP*_, HP variance; μ_*SAP*_, SAP mean; σ^2^_*SAP*_, SAP variance; μ_*DAP*_, DAP mean; σ^2^_*DAP*_, DAP variance. The symbol ^∗^ indicates *p* < 0.05 versus PRE within the same group (i.e., noAKD or AKD). The symbol §  indicates *p* < 0.05 versus noAKD within the same experimental condition (i.e., PRE or POST).*

As expected, *K*^2^ and BRS markers were computed in 100% of the subjects via AVG and MAX strategies. Conversely, it was possible to compute *K*^2^ and BRS markers with the WCF strategy in the 96% of noAKD subjects during PRE, 99% of noAKDs during POST, 93.1% of AKDs during PRE and 100% of AKDs during POST. BRS_SAP__→__HP_ and a_HP__→__SAP_(1) were computed in 100% of the subjects irrespective of the group.

Table 3 shows the results of *K*^2^ assessed between HP and SAP according to the different strategies (i.e., MAX, AVG, and WCF). All *K*^2^ markers were reduced during POST compared to PRE. Reduction was significant regardless of the group with the notable exception of *K*^2^_AVG_ that diminished significantly only in noAKDs. None of the *K*^2^ markers was able to differentiate the two groups.

**TABLE 3:** *K*^2^ values computed according to the three different strategies.

**Marker**	**PRE**		**POST**	
	**noAKD**	**AKD**	**noAKD**	**AKD**
*K*^2^_MAX_	0.56 ± 0.22	0.56 ± 0.23	0.33 ± 0.19^∗^	0.28 ± 0.16^∗^
*K*^2^_WCF_	0.36 ± 0.39	0.30 ± 0.42	0.17 ± 0.16^∗^	0.12 ± 0.10^∗^
*K*^2^_AVG_	0.35 ± 0.20	0.36 ± 0.19	0.16 ± 0.11^∗^	0.12 ± 0.08

*HP, heart period; SAP, systolic arterial pressure; LF, low frequency; *K*^2^, squared coherence between HP and SAP; *K*^2^_*MAX*_, maximum *K*^2^ in the LF band; *K*^2^_*AVG*_, averaged *K*^2^ in the LF band; K^2^_*WCF*_, *K*^2^ sampled at the weighted central frequency of the SAP spectral components in the LF band. The symbol ^∗^ indicates *p* < 0.05 versus PRE.*

**FIGURE 2:**
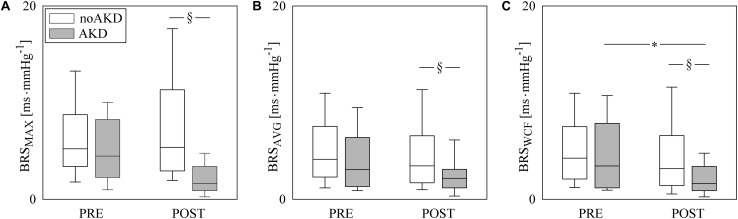
The box-and-whisker graphs show BRS_MAX_ **(A)**, BRS_AVG_ **(B)**, and BRS_WCF_ **(C)** as a function of the experimental condition (i.e., PRE and POST) in noAKD (white boxes) and AKD (gray boxes) individuals. Box height represents the interquartile range, median is marked with a solid line and whiskers denote the 5th and 95th percentile. The symbol ^∗^ indicates a significant change between experimental conditions (i.e., PRE and POST) within the same group (i.e., noAKD or AKD), while the symbol §  indicates a significant difference between groups within the same experimental condition with *p* < 0.05.

Box-and-whisker plots of Figure 2 show the BRS values as a function of the experimental condition (i.e., PRE and POST) in noAKDs (white boxes) and AKDs (gray boxes). The BRS estimates are computed via the MAX (Figure 2A), AVG (Figure 2B), and WCF (Figure 2C) strategies. BRS_MAX_, BRS_AVG_ and BRS_WCF_ were significantly reduced during POST in AKD subjects compared to noAKD ones, while no between-group differences were observed during PRE. BRS_MAX_ and BRS_AVG_ were not affected by propofol anesthesia regardless of the group (i.e., noAKD or AKD). Conversely, BRS_WCF_ decreased during POST in AKD group, while it was not influenced by propofol anesthesia in noAKDs.

**FIGURE 3:**
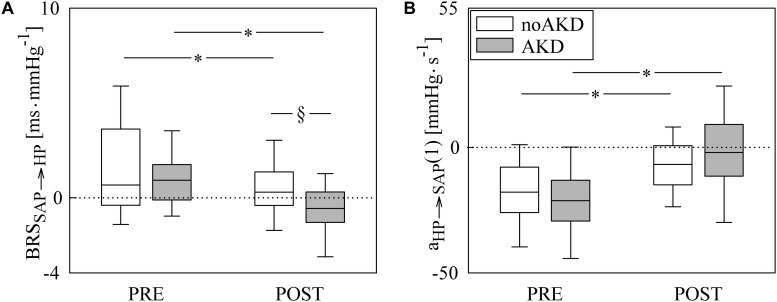
The box-and-whisker graphs show BRS_SAP__→__HP_ **(A)** and a_HP__→__SAP_(1) **(B)** as a function of the experimental condition (i.e., PRE and POST) in noAKD (white boxes) and AKD (gray boxes) individuals. Box height represents the interquartile range, median is marked with a solid line and whiskers denote the 5th and 95th percentile. The dotted lines mark the zero value. The symbol ^∗^ indicates a significant change between experimental conditions (i.e., PRE and POST) within the same group (i.e., noAKD or AKD), while the symbol §  indicates a significant difference between groups within the same experimental condition with *p* < 0.05.

Figure 3 has the same structure of Figure 2 but it shows results of BRS_SAP__→__HP_ (Figure 3A) and a_HP__→__SAP_(1) (Figure 3B). BRS_SAP__→__HP_ was reduced during POST with respect to PRE regardless of the group (i.e., noAKD or AKD). Moreover, after the induction of anesthesia BRS_SAP__→__HP_ in AKDs was lower than in noAKDs. The effect of the anesthesia was evident over a_HP__→__SAP_(1): indeed, it became less negative during POST in both groups. However, a_HP__→__SAP_(1) was not able to differentiate noAKDs from AKDs regardless of the experimental condition (i.e., PRE or POST).

**FIGURE 4:**
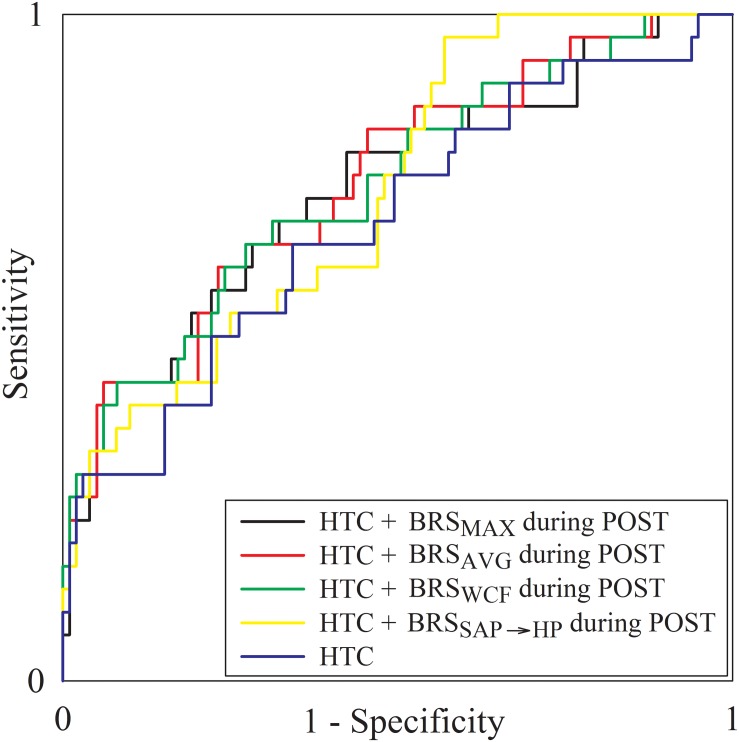
The multiple line plot shows the ROC curves obtained from the multivariate logistic regression models built using only HTC (blue line) and by combining HTC with BRS_SAP__→__HP_ during POST (yellow line), HTC and BRS_WCF_ during POST (green line), HTC with BRS_AVG_ during POST (red line), and HTC with BRS_MAX_ during POST (black line).

All the indexes exhibiting between-group differences at a univariate level (i.e., σ^2^_HP_ and σ^2^_SAP_ during PRE, BRS_MAX_, BRS_AVG_, BRS_WCF_, and BRS_SAP__→__HP_ during POST) entered a multivariate logistic regression model accounting for clinical and demographic factors resulting different between noAKD and AKD groups with a *p* < 0.1 (i.e., HTC and preoperative serum creatinine level). Only BRS indexes, regardless of the strategy used to calculate them, remained significantly associated with the outcome while time domain indexes did not. Moreover, when BRS markers were examined in combination with HTC and preoperative serum creatinine level, only HTC remained associated to the outcome. Regression coefficient, odds ratio, 95% confidence interval and type I error probability *p* of the multivariate logistic regression models are shown in Table 4 as well as AUCs of the ROC curves. It can be observed that the combination of clinical parameters (i.e., HTC) with the BRS improves the predictive power of AKD as stressed by the increase of AUC compared to the model accounting for the sole HTC. However, the difference between the AUC computed over HTC and that computed by combining HTC with BRS markers is limited. It is worth noting that the model combining HTC with BRS_AVG_ during POST achieved the highest AUC at multivariate level. Figure 4 shows the superposition of the ROC curves assessed from the multivariate logistic regression models built using only HTC (blue line) and by combining HTC with BRS_SAP__→__HP_ during POST (yellow line), HTC and BRS_WCF_ during POST (green line), HTC with BRS_AVG_ during POST (red line), and HTC with BRS_MAX_ during POST (black line).

**TABLE 4:** Results of multivariate logistic regression analysis for AKD prediction.

**Parameter**	**Regression coefficient**	**Odds ratio**	**95% confidence interval**	***p***	**AUC**
BRS_MAX_ during POST	−0.118	0.889	0.798–0.991	0.034	0.741
HCT	−0.196	0.822	0.731–0.925	0.001	
Constant	6.870	963.067		0.002	
BRS_AVG_ during POST	−0.188	0.829	0.697–0.984	0.032	0.747
HCT	−0.194	0.823	0.732–0.926	0.001	
Constant	6.823	918.982		0.003	
BRS_WCF_ during POST	−0.154	0.857	0.736–0.998	0.048	0.742
HCT	−0.203	0.817	0.725–0.920	0.001	
Constant	7.017	531.34		0.002	
BRS_SAP__→__HP_ during POST	−0.345	0.708	0.529–0.948	0.020	0.731
HCT	−0.184	0.832	0.741–0.934	0.002	
Constant	5.687	295.054		0.010	
HCT	−0.200	0.819	0.731–0.918	0.001	0.690
Constant	6.344	568.82		0.004	

*BRS, baroreflex sensitivity; HP, heart period; SAP, systolic arterial pressure; LF, low frequency; BRS_*MAX*_, BRS obtained by sampling the transfer function gain between HP and SAP at the maximum of HP-SAP squared coherence in LF band; BRS_*AVG*_, BRS obtained as the average of the transfer function gain between HP and SAP in LF band; BRS_*WCF*_, BRS obtained by sampling the transfer function gain between HP and SAP at the weighted central frequency of the SAP spectral components in LF band; BRS_*SAP*__→__*HP*_, causal closed loop BRS; POST, after anesthesia induction; HCT, hematocrit; AUC, area under the receiver operating characteristic curve.*

Receiver operating characteristic curves were also calculated using solely BRS markers able to distinguish AKD and noAKD groups (i.e., BRS_MAX_, BRS_AVG_, BRS_WCF_, and BRS_SAP__→__HP_). The AUC of ROC curves relevant to BRS_MAX_, BRS_AVG_, BRS_WCF_, and BRS_SAP__→__HP_ during POST were 0.641, 0.662, 0.658, and 0.671 respectively. The cutoff value for each BRS marker was calculated according to the Youden’s index, and the corresponding sensitivity, specificity, PPV and NPV were computed and reported in Table 5. It can be observed that BRS_SAP__→__HP_ reported the highest AUC assessed at the univariate level with the highest specificity (i.e., 79%) and PPV (i.e., 41.7%) compared to BRS_MAX_, BRS_AVG_, and BRS_WCF_. The improved specificity and PPV of BRS_SAP__→__HP_ were reached at the cost of a reduced sensitivity (i.e., 51.7%) and NPV (i.e., 84.9%) with respect to BRS_MAX_, BRS_AVG_, and BRS_WCF_.

**TABLE 5:** Results of logistic regression analysis for AKD prediction.

**Parameter**	**cutoff [ms⋅mmHg^–1^]**	**sensitivity**	**specificity**	**PPV**	**NPV**	**AUC**
BRS_MAX_ during POST	7.73	93.1%	36.4%	29.8%	94.8%	0.641
BRS_AVG_ during POST	3.17	82.8%	53.5%	34.1%	91.5%	0.662
BRS_WCF_ during POST	4.82	93.1%	28.5%	29.5%	94.6%	0.658
BRS_SAP__→__HP_ during POST	−0.59	51.7%	79.0%	41.7%	84.9%	0.671

*BRS, baroreflex sensitivity; HP, heart period; SAP, systolic arterial pressure; LF, low frequency; BRS_*MAX*_, BRS obtained by sampling the transfer function gain between HP and SAP at the maximum of HP-SAP squared coherence in LF band; BRS_*AVG*_, BRS obtained as the average of the transfer function gain between HP and SAP in LF band; BRS_*WCF*_, BRS obtained by sampling the transfer function gain between HP and SAP at the weighted central frequency of the SAP spectral components in LF band; BRS_*SAP*__→__*HP*_, causal closed loop BRS; POST, after anesthesia induction; PPV, positive predictive value; NPV, negative predictive value; AUC, area under the receiver operating characteristic curve.*

## Discussion

The main findings of this work can be summarized as follows: (i) propofol general anesthesia depresses autonomic function and cardiovascular control; (ii) the reduction of BRS during propofol general anesthesia is more evidently detected using causal than non-causal BRS estimates; (iii) time domain markers are weakly associated with AKD; (iv) BRS markers can separate AKD from noAKD individuals regardless of the computational strategy; (v) BRS assessed after propofol general anesthesia induction is reduced in subjects developing AKD; (vi) both non-causal and causal BRS markers remain associated to AKD even after accounting for clinical and demographic confounding factors; (vii) performances of non-causal and causal BRS indexes in stratifying the risk of AKD after CABG were similar.

### Autonomic Function and Cardiovascular Control Are Depressed During Propofol General Anesthesia

Propofol-based general anesthesia is known to depress autonomic function and cardiovascular control (Boer et al., 1990; Ebert et al., 1992; Sellgren et al., 1994; Hidaka et al., 2005; Sato et al., 2005; El Beheiry and Mak, 2013; Porta et al., 2013) leading to bradycardia (Tramer et al., 1997) and hypotension (Au et al., 2016). This result is confirmed in this study. Indeed, the mean of HP increased and mean of both SAP and DAP decreased. Moreover, the decrease of HP variance suggests a depression of autonomic control (Pomeranz et al., 1985). The effect of propofol general anesthesia was less strong on variance of SAP than HP and that on DAP variance was even weaker. The effect of propofol anesthesia was more evident over causal than non-causal BRS markers: indeed, the causal BRS marker was the sole index able to indicate the decrease of BRS in both AKDs and noAKDs during POST. The better performance of the causal closed loop BRS estimate compared to non-causal BRS markers in detecting the impairment of the BR control during propofol anesthesia was first suggested over a smaller group of CABG patients in Porta et al. (2013). The improved performance is likely to be due to the ability of the causal closed loop approach to account for the non-baroreflex-mediated origin of part of the HP variability in the LF band (Preiss and Polosa, 1974; Baselli et al., 1994) and for the anticausal effects related to the active presence of mechanical feedforward pathway (Porta et al., 2013). Also the migration of the gain of the mechanical feedforward arm toward 0 during propofol anesthesia, originally observed in Porta et al. (2013) and interpreted along with the BRS decrease as an indication of the depression of the overall HP-SAP control loop, was confirmed in the present study. The reduction of the impact of HP variability on SAP changes along the mechanical feedforward pathway is the likely consequence of vasodilatation and reduced left ventricular contractility induced by propofol anesthesia (Porta et al., 2013). The overall depression of the cardiovascular control in response to propofol anesthesia was suggested even by the significant decrease of *K*^2^ during POST regardless of the method utilized for the computation of *K*^2^ marker.

### Time Domain Markers Separate AKDs From noAKDs but They Are Not Associated With AKD After Accounting for Clinical and Demographic Factors

Few markers in the time domain were able to separate AKD and noAKD groups during PRE (i.e., σ^2^_HP_ and σ^2^_SAP_). Since σ^2^_HP_ is directly linked to the amplitude of both vagal and sympathetic outflow modulations directed to the heart (Pomeranz et al., 1985; Montano et al., 1994), while σ^2^_SAP_ raises with the relevance of sympathetic outflow modulations directed to the vessels (Pagani et al., 1997; Cooke et al., 1999; Marchi et al., 2016a), it can be hypothesized that autonomic regulation could play a role in AKD development. Conversely, none of the time domain parameters during POST was able to differentiate AKD from noAKD group and this limited ability can be taken again a hallmark of the autonomic function depression following propofol general anesthesia. However, when σ^2^_HP_ and σ^2^_SAP_ during PRE were added one by one to clinical and demographic markers featuring the best performance in separating AKDs from noAKDs (i.e., HTC and serum creatinine level), time domain markers did not provide complementary information, thus supporting the concept that AKD is weakly linked to the magnitude of autonomic activity and/or modulation. This disappointing finding could be the consequence of the unspecific characteristic of these two markers, thus prompting for the exploitation of cardiovascular control indexes that are much more specifically linked to regulatory reflexes such as the BR.

### BRS Is Lower in AKDs During POST and the BRS Association With AKD Remains After Accounting for Clinical and Demographic Factors

Both non-causal and causal BRS estimates were able to distinguish AKD and noAKD groups while markers measuring the mere association between HP and SAP variability series (i.e., *K*^2^ indexes) and the gain of the mechanical feedforward pathway were useless. Remarkably, all BRS indexes remained associated to the adverse outcome even when combined with clinical and demographic parameters that were detected to be significantly associated to AKD, thus confirming the relevance of the association between BRS and AKD (Ranucci et al., 2017). Remarkably, this association was confirmed in this study using an additional class of methods for BRS estimation, namely the causal closed loop method. More specifically, we found that BRS was lower in patients who developed AKD after CABG, thus suggesting that a more active BR control is protective against AKD, while a depressed BR regulation should be considered a risk factor for the development of AKD. The association between low BRS values and AKD could be the consequence of an insufficient BR response to hypotension and hypoperfusion situations that might be occurred during CABG surgery (Mangano et al., 1998; Provenchere et al., 2003; Pavlov and Tracey, 2012). However, given the link of a low BRS with vagal withdrawal and sympathetic activation (Cooke et al., 1999; Marchi et al., 2016b; De Maria et al., 2018), the association between a depressed BRS value and AKD could be the result of the abnormal reaction to inflammation and anomalous levels of oxidative stress favored by a limited vagal control and a high sympathetic drive (Pavlov and Tracey, 2012; Inoue et al., 2016a, b). Our interpretation of the findings privileges the causal pathway that an impaired BR causes AKD. This interpretation is supported by the lower BRS observed during POST well before the AKD development and by the observation that an impaired autonomic control is a risk factor for chronic kidney disease (Brotman et al., 2010). However, the reverse pathway, namely an incoming, or manifest, AKD could determine a BR impairment, cannot be dismissed. Indeed, previous studies have shown how the renal impairment can be an independent risk factor for cardiovascular events and heart failure (Omar and Zedan, 2013) and that an increased afferent renal sympathetic activity from impaired kidneys is critically involved in the pathogenesis of sympathetic hyperactivity (Blankestijn and Joles, 2012).

Remarkably, the association of BRS markers with the outcome was observed during POST, while it was not visible during PRE. This result might be at first sight quite surprising given the well-known depression of autonomic control and baroreflex regulation during propofol general anesthesia (Boer et al., 1990; Ebert et al., 1992; Sellgren et al., 1994; Hidaka et al., 2005; Sato et al., 2005; El Beheiry and Mak, 2013; Porta et al., 2013) confirmed even by the present study. However, on the one hand, baroreflex regulation is depressed but not absent (Porta et al., 2013) and, on the other hand, hemodynamic instability observed during CABG surgery, brief periods of inadequate delivery of oxygen to the tissues (Toner et al., 2016), and episodes of hypotension and hypoperfusion (Ranucci et al., 2017) might have stimulated more importantly the residual BR control during POST than PRE. Also mechanical ventilation, profoundly influencing venous return and stroke volume, might have contributed to solicit the residual BR at frequencies slower than the ventilatory one.

We point out that the cutoff values of BRS assessed according to the Youden’s index varies with the strategy for BRS computation, being equal to 7.73, 3.17, 4.82, and −0.59 ms⋅mmHg^–1^ for BRS_MAX_, BRS_AVG_, BRS_WCF_, and BRS_SAP__→__HP_ respectively. The results relevant to non-causal BRS estimates are in agreement with the different cutoff values of BRS utilized to predict adverse events present in literature (La Rovere et al., 1998; Gouveia et al., 2015; Toner et al., 2016; Ranucci et al., 2017; Pinna et al., 2017). Cutoff values depend on the method exploited for BRS estimation (Pinna et al., 2017), type of pathology (La Rovere et al., 1998; Gouveia et al., 2015) and endpoint of the analysis (Toner et al., 2016; Ranucci et al., 2017). This study suggests that even within the same class of methods for the BRS estimation (i.e., the non-causal class in the frequency domain) cutoff values might vary importantly according to the strategy followed to derive the final BRS values utilized to typify the patient. Since BRS_SAP__→__HP_ was computed according to a completely different method (i.e., the causal closed loop technique), it is not surprising that the cutoff value of BRS_SAP__→__HP_ was significantly different from those of the non-causal markers. Indeed, it was negative according to the possibility given by its definition computing the slope of the HP response to an artificial SAP rise (Baselli et al., 1994). A negative BRS_SAP__→__HP_ implies that, instead of having a bradycardic response to a SAP rise, a tachycardic reaction is observed. This result provides a new perspective on the BR control in CABG patients: indeed, the patients most at risk of developing AKD after CABG are not simply those who have the most depressed BR control but those who exhibit an antiparallel HP response to SAP variations. An additional factor that might increase the variability of the cutoff is the experimental condition during which the signals were recorded. For example, BRS estimates computed in this study were useless in separating AKD from noAKD individuals when evaluated during PRE. These considerations stress the need for a standardization of the BRS assessment to favor future clinical applications especially whether BRS methods based on spontaneous variability will be exploited. However, roughly speaking about non-causal frequency domain BRS markers, we confirmed that a cutoff value of about 3 ms⋅mmHg^–1^ can be utilized as a first attempt to make prediction of adverse outcomes in clinical context where BRS estimates are found to be significantly associated to the event (La Rovere et al., 1998). However, this value does not hold for causal closed loop BRS estimates.

### Comparison of the Performances of Non-causal and Causal BRS Estimates in Stratifying the Risk of AKD in CABG Patients

The present study originally compares the performance of non-causal and causal BRS markers in stratifying the risk of AKD after CABG. Performances were similar regardless of the class of method (i.e., non-causal or causal class) and within the non-causal class irrespective of the strategy followed to derive a unique index from the transfer function gain in LF band. It can be observed that the BRS_AVG_ is slightly superior in terms of AUC at multivariate level (i.e., in combination with HTC) and the presumed superiority of BRS_SAP__→__HP_ compared to non-causal BRS indexes, resulting from its more complex model structure, was evident only at univariate level. The BRS_AVG_ should be preferred given the simplicity of its computation and remarkable performance. Indeed, the BRS_AVG_ does not require the presence of a spectral peak in the LF band in the SAP series like the BRS_*W*__*CF*_, the presence of a *K*^2^ peak in the LF band like the BRS_MAX_, and the identification of a complex model structure including RESP like the BRS_SAP__→__HP_. This study confirms the feeling pointed out in Pinna et al. (2017) that in practical applications the full adherence to the concept of the BR, undoubtedly provided by the causal closed loop strategy, does not assure a measurable advantage compared to simpler non-causal techniques likely due to the complexity of the physiological interactions and the effect of noise on the final estimate making alike the performance of all the considered methods.

### Limitations of the Study and Future Developments

A limitation of this study is the impossibility of testing the ability of BRS in discriminating acute kidney injury (i.e., the increase of postoperative creatinine of more than 50% with respect to preoperative level), that is a more stringent and risky condition with respect to AKD. This limitation is due to the relatively small number of patients who developed acute kidney injury in our database that prevented any reliable association of BRS markers with this adverse event. The application of the same approach after the enlargement of the enrolled population would allow us in the future to test the association of BRS markers with acute kidney injury as well.

Moreover, in interpreting the results we privilege the possibility that a reduced BRS could play a role in the post-surgery development of AKD but the action of the reverse pathway, implying a potential influence of an incoming AKD on BR control, cannot be fully dismissed and calls for additional studies.

## Conclusion

This work stresses the relevance of computing BRS markers from spontaneous variability of HP and SAP computed via causal and non-causal methods to stratify the risk of AKD after CABG and their complementary information compared to clinical and demographic factors. Remarkably, the present study provided cutoff values that are worth being exploited to identify subjects at risk of AKD after CABG. Moreover, we conclude that no evident clinical improvement can be derived from the application of causal BRS markers with respect to non-causal BRS ones. Future studies should verify the clinical impact of the application of the provided cutoff values and should test whether the use of premedications and/or countermeasures before CABG surgery aiming at increasing BRS might have a favorable impact on the incidence of postoperative AKD.

## Data Availability Statement

The datasets generated for this study are available on request to the corresponding author.

## Ethics Statement

The study was performed in keeping with the Declaration of Helsinki for research studies involving humans and, before participating, subjects signed a written informed consent. The study was approved by the ethical committee in charge at the IRCCS Policlinico San Donato, San Donato Milanese, Milan, Italy.

## Author Contributions

VB and AP contributed to the conception and design of research. VB, EV, VP, AF, and MR performed the experiments. VB, EV, BC, and BD analyzed the data. VB and AP drafted the manuscript and prepared the figures. VB, EV, VP, AF, BC, BD, LD, MR, and AP interpreted the results, edited and revised critically the manuscript, and approved the final version of the manuscript.

## Conflict of Interest

The authors declare that the research was conducted in the absence of any commercial or financial relationships that could be construed as a potential conflict of interest. The reviewer MJ declared a past co-authorship with one of the authors AP to the handling Editor.
